# Changing dynamics of caregiving: a meta-ethnography study of informal caregivers’ experiences with older immigrant family members in Europe

**DOI:** 10.1186/s12913-023-09023-4

**Published:** 2023-01-17

**Authors:** Sunita Shrestha, Sanjana Arora, Alistair Hunter, Jonas Debesay

**Affiliations:** 1 Department of Nursing and Health Promotion, Faculty of Health Sciences, Oslo Metropolitan University, Oslo, Norway; 2 Centre for Intercultural Communication, VID Specialized Univeristy, Stavanger, Norway; 3 School of Interdisciplinary Studies, University of Glasgow, Dumfries, UK

**Keywords:** Family caregiver, Next of kin, Immigrant, Ageing, Informal care, Experience, Qualitative research

## Abstract

**Background:**

The population of Europe is ageing and becoming more ethnically diverse due to migration. Finding suitable long-term caring arrangements for older immigrants in Europe has been one of healthcare policymakers’ concerns in the last decade. However, relatively few older people with an immigrant background live in long-term care facilities, and many prefer to be cared for by their family members. Little is known about immigrant family caregivers’ experiences of caring for older family members and the support they need while providing care. This study aims to synthesize the qualitative literature exploring the experiences of individuals caring for older family members with immigrant backgrounds from Africa, Asia and South America living in Europe.

**Methods:**

We searched the electronic databases Medline Ovid, Embase Ovid, PsycInfo Ovid, SocIndex EBSCOhost, CINAHL EBSCOhost, Scopus, Social Care Online, ASSIA ProQuest, and Google Scholar for original, peer reviewed research articles, published in English from 2011 to 2022. The seven-step interpretive methodology in meta-ethnography developed by Noblit and Hare (1988) was followed for qualitative synthesis.

**Results:**

After assessing 4155 studies for eligibility criteria, 11 peer-reviewed articles were included in this review. The qualitative synthesis of these included articles resulted in four main themes: strong care norms for parents, the moral dilemma of continuing care, uneven care sharing, and the use of formal care services.

**Conclusions:**

Caregiving dynamics are changing, both in terms of motivations and approaches to caregiving. Furthermore, there are gender disparities in the distribution of caregiving duties, particularly with women carrying the more significant burden of care. The care burden is further exacerbated by the lack of culturally sensitive formal services complementing the care needs of the ageing immigrants and their family caregivers. Therefore, those searching for alternatives to informal care should be met with appropriate health and care services in terms of language, culture, religion, and lifestyle, delivered in a non-judgmental way.

**Supplementary Information:**

The online version contains supplementary material available at 10.1186/s12913-023-09023-4.

## Background

The population of Europe is ageing and becoming more ethnically diverse due to migration [1]. Europe has experienced significant flows of refugees and immigrants from non-European countries since the late 2000s [1]. Though many immigrants are young adults, the number of older immigrants is increasing [1, 2]. As people age, they have more significant healthcare needs than the general population [3–5]. They are, e.g., more likely to experience multimorbidity [6] and are prone to several chronic conditions such as; hearing loss, cataracts and refractive errors, back and neck pain and osteoarthritis, chronic obstructive pulmonary disease, diabetes, depression, dementia, cancer or stroke [7]. Particularly among non-European older immigrants, self-rated health, well-being, and mental health status tend to be lower than the majority population, with steeper health decline in later life [1].

Finding suitable long-term caring arrangements for older immigrants in Europe has been one of healthcare policymakers’ concerns in the last decade [8, 9]. However, ageing immigrants often have poor access to healthcare due to their lower socioeconomic status [10, 11], limited health information on available services [11], limited language skills [12], difficulty in navigating health systems [13], as well as a lack of cultural sensitivity within some health services [14]. In addition, relatively few older people with an immigrant background live in long-term care facilities such as nursing homes [1, 15] and many prefer to be cared for by their family members [1, 16–18]. Furthermore, the capacity of family members to assume caregiving roles is often shaped by cultural norms, filial obligations [2, 18, 19], and gender roles [16, 20, 21].

In general, among both immigrant and non-immigrant populations, family caregivers such as family members, friends, and neighbours provide as much as 90% of in-home long-term care needed by older adults [22], and the primary caregiving responsibilities remain largely with the female family members [20]. There is uncertainty about family caregivers’ ability and willingness to assume the full responsibility of caregiving, although most children feel responsible and are motivated to care for their older relatives [2, 13].The experiences of providing care also tend to vary between different generations [2, 23, 24]. Caregiving to older adults enhances family caregivers’ personal growth, self-efficacy, satisfaction, appreciation of life and closer relationship with care receivers [25, 26]. Nevertheless, family caregivers of older adults have been found vulnerable to poor health outcomes from physical challenges to chronic stress, anxiety, feelings of guilt, and embarrassment [12, 13, 16, 27, 28], as well as social isolation and limited social networks [13] due to overwhelming caregiving burden.

There is a need to identify the experiences of family caregivers with an immigrant background to understand how better support can be provided to those caring for an older relative, thereby achieving better cooperation between family caregivers and formal care services. However, there is still limited research on the issue of informal caregiving among immigrants. Research in the area of caregiving within immigrant families mostly addresses the caregiving of older adults with dementia [12, 13, 29–31]. There is no review study so far addressing the experiences of immigrant-background family caregivers who are caring for older relatives with no specific diagnosis or diseases other than dementia. Thus, this review study aims to synthesize the qualitative literature exploring the experiences of individuals with an immigrant background in caring for older family members from Africa, Asia and South America living in Europe. It emphasizes understanding the meaning of caregiving for family members from different generations and how caregiving has been negotiated in their day-to-day life.

## Methods

In this study we use meta-ethnography developed by Noblit and Hare (1988), a seven-step interpretive methodology for qualitative synthesis [32] which is a widely used approach in health and social care [33]. It facilitates interpretative synthesis by systematically comparing conceptual data from primary qualitative research to identify and develop new overarching concepts, theories, and models by preserving the original meanings and contexts of study concepts [32]. The seven-step analysis includes the following phases: 1) getting started, 2) deciding what is relevant to the initial interest, 3) reading the studies, 4) determining how the studies are related, 5) translating studies into one another, 6) synthesizing the translations and 7) expressing the synthesis. The process is iterative and the phases may occur in a less linear way [32]. We followed the operationalized guidelines developed by Sattar et al. [34], based on the original seven steps as developed by Noblit & Hare [34].

### Phase 1: getting started

The four researchers (SS, SA, AH and JD) from the interdisciplinary field of social sciences and nursing teamed up for this review study to synthesize studies based on the research question “What are the experiences of individuals with an immigrant background in providing care to their older family members in Europe.” This systematic review was registered with PROSPERO (reg, no CRD42022340285).

### Phase 2: deciding what is relevant to the initial interest

After deciding on the research question and the possible keywords, we collaborated with librarians at OsloMet University to refine the literature search. The inclusion/exclusion criteria were developed both before and during the search. Based on the aim of the study, we agreed on including keywords such as dementia, family caregiver, next of kin, immigrant, ageing, informal care, experience and qualitative research. We conducted a systematic literature search of eight electronic databases: Medline Ovid, Embase Ovid, PsycInfo Ovid, SocIndex EBSCOhost, Cinahl EBSCOhost, Scopus, Social Care Online, ASSIA ProQuest and Google Scholar during August–September 2021. An example of the full electronic search strategy in the CINAHL database is presented in Table 1 (See supplementary material 1 and 2 for search strategies in other databases).

**Table 1:** Search strategy in database CINAHL

Date: 28.09.2021		
Results: 1733		
**#**	**Query**	**Results**
**S1**	(MH “Aged”) OR (MH “Aged, 80 and Over”) OR (MH “Frail Elderly”)	881,663
**S2**	(MH “Dementia”) OR (MH “Dementia Patients”)	44,371
**S3**	(MH “Geriatrics”)	5,733
**S4**	TI ( ((aged or old*) N1 (people or women or person* or men or immigrant* or minorit* or parent*))) OR AB ( ((aged or old*) N1 (people or women or person* or men or immigrant* or minorit* or parent*)))	95,630
**S5**	TI ( (senior or seniors or geriatric or elder or elders or elderly or dement* or aging or ageing or “old age”)) OR AB ( (senior or seniors or geriatric or elder or elders or elderly or dement* or aging or ageing or “old age”))	250,262
**S6**	TI old*	103,533
**S7**	S1 OR S2 OR S3 OR S4 OR S5 OR S6	1,058,844
**S8**	(MH “Caregivers”) OR (MH “Caregiver Burden”) OR (MH “Caregiver Attitudes”) OR (MH “Caregiver Support”)	48,117
**S9**	TI ( ( caregiver* or “care giver*” or caretaker* or “care taker*” or carer*)) OR AB ( ( caregiver* or “care giver*” or caretaker* or “care taker*” or carer*))	70,794
**S10**	(MH “Adult Children”) OR (MH “Family”) OR (MH “Family Relations”) OR (MH “Nuclear Family”) OR (MH “Daughters”) OR (MH “Siblings”) OR (MH “Sons”) OR (MH “Spouses”) OR (MH “Parents”)	119,876
**S11**	(MH “Patient-Family Relations”) OR (MH “Fathers”) OR (MH “Mothers”) OR (MH “Significant Other”)	40,247
**S12**	S10 OR S11	153,957
**S13**	(MH “Caring”)	8,871
**S14**	AB ( caring or caregiving or (care N1 giving))	40,743
**S15**	S13 OR S14	46,740
**S16**	S12 AND S15	7,417
**S17**	TI ( caring or caregiving or (care N1 giving))	20,213
**S18**	TI ( ((children* or son or sons or daughter* or offspring* or sibling* or brother* or sister* or wife* or wives or husband* or partner* or spous* or married* or famil* or parent* or father* or mother* or “next of kin*” or kinship* or “significant other*” or relative or relatives or informal or unpaid or old* or elder* or aged or aging or ageing or senior or seniors or geriatric) N2 (care or caring or caregiving))) OR AB ( ((children* or son or sons or daughter* or offspring* or sibling* or brother* or sister* or wife* or wives or husband* or partner* or spous* or married* or famil* or parent* or father* or mother* or “next of kin*” or kinship* or “significant other*” or relative or relatives or informal or unpaid or old* or elder* or aged or aging or ageing or senior or seniors or geriatric) N2 (care or caring or caregiving)))	75,458
**S19**	S8 OR S9 OR S16 OR S17 OR S18	160,895
**S20**	(MH “Immigrants”) OR (MH “Immigrants, Illegal”) OR (MH “Emigration and Immigration”)	21,404
**S21**	(MH “Minority Groups”) OR (MH “Ethnic Groups”) OR (MH “Cultural Diversity”) OR (MH “Cultural Values”)	59,887
**S22**	(MH “Transients and Migrants”) OR (MH “Undocumented Immigrants”) OR (MH “Refugees”)	13,613
**S23**	TI ( ( Immigrant* or migrant* or multicultur* or intercultur* or minorit* or ethnic* or multiethnic* or racial* or refugee* or “non western*” or nonwestern* or “asylum seeker*”)) OR AB ( ( Immigrant* or migrant* or multicultur* or intercultur* or minorit* or ethnic* or multiethnic* or racial* or refugee* or “non western*” or nonwestern* or “asylum seeker*”))	127,448
**S24**	S20 OR S21 OR S22 OR S23	169,809
**S25**	(MH “Qualitative Studies”) OR (MH “Action Research”) OR (MH “Ethnographic Research”) OR (MH “Ethnological Research”) OR (MH “Ethnonursing Research”) OR (MH “Grounded Theory”) OR (MH “Naturalistic Inquiry”) OR (MH “Phenomenological Research”) OR (MH “Phenomenology”) OR (MH “Focus Groups”) OR (MH “Narratives”) OR (MH “Interviews”) OR (MH “Semi-Structured Interview”) OR (MH “Structured Interview”) OR (MH “Unstructured Interview”)	322,392
**S26**	(MH “Attitude”) OR (MH “Family Attitudes”) OR (MH “Caregiver Attitudes”) OR (MH “Behavior”) OR (MH “Perception”)	75,106
**S27**	TI ( ( Qualitative or Narrative or Phenomenolog* or Hermeneutic* or interview* or “Grounded theor*” or Ethnograph* or themes or Attitude* or Behavior* or Behaviour* or Perception* or View* or experience* or “focus group*”)) OR AB ( ( Qualitative or Narrative or Phenomenolog* or Hermeneutic* or interview* or “Grounded theor*” or Ethnograph* or themes or Attitude* or Behavior* or Behaviour* or Perception* or View* or experience* or “focus group*”))	1,125,368
**S28**	S25 OR S26 OR S27	1,234,025
**S29**	S7 AND S19 AND S24 AND S28	1,733

The International Organization for Migration (IOM) report of 2006 on European Research calls for more research on migration and health, making it likely to have more relevant research articles on this topic in subsequent years [35]. To maximize coverage, the initial search was performed without any limitation regarding the year of publication, which covered publications through the end of September 2021 and had 6069 hits via database search. To retrieve more relevant research in line with IOM’s report [35], along with considerations about the scope and timeframe of the study, we narrowed the search to include only studies published between 2011 and 2021 and later updated on 30^th^ June 2022 to capture the most recent relevant publications. Further we limited studies to participants with immigrant backgrounds from Asia, Africa and South America as they tend to have a higher risk of poverty, lower levels of education [36] and are most disadvantaged in terms of health inequalities [10, 23, 37].

**Fig. 1:**
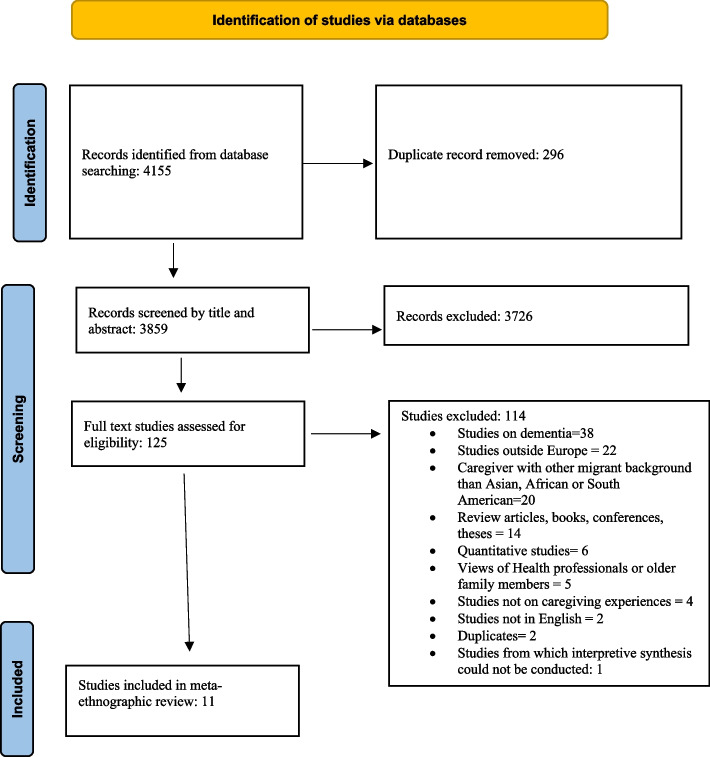
PRISMA flow diagram of study selection

We obtained 4155 hits via database search, and the references were transferred to the reference management software EndNote (version X9). These references were additionally uploaded to the systematic review management software Covidence [38], and it removed 296 duplicates. All authors were involved in screening tittles and abstracts, and each paper was screened by two authors independently, based on the inclusion/exclusion criteria (Table 2). We extracted 125 articles for a full-text review. Two qualitative systematic review studies [12, 13] on caregivers’ experience with older adults with dementia were published in November 2021 and June 2022 respectively, after our literature search. We therefore decided to exclude studies on dementia, though dementia was originally one of the search elements in the literature search. Studies which focused on participants’ experiences of caregiving for older adults aged 50 years or over, or those which described care receivers as older adults were included. This is also in line with WHO’s inclusion criterion of old age for research on participants’ from low income countries such as in the African context [39]. Further, only studies conducted in European countries were included due to similar historical development of migration from Asia, Africa and South America to the main European destination countries [1]. Finally, 11 peer-reviewed articles were selected. The study selection process is presented in the PRISMA flow diagram below (Fig. 1). To assess the methodological quality of the 11 studies, the four authors independently assessed the Critical Appraisal Skills Program’s (CASP) [40] qualitative checklist before including them for further analysis (Table 3).

**Table 2:** Overview of selection criteria for meta-synthesis

Criteria	Inclusion	Exclusion
Study design	• Qualitative studies or mixed methods studies with individual interviews and/or focus groups	• Quantitative studies • Mixed methods studies in which qualitative findings cannot be separated from the quantitative studies • Qualitative studies where informal caregivers’ views cannot be separated from other study participants’ views • Qualitative studies where immigrant or non-immigrant background is not possible to identify • Qualitative studies with ‘thin descriptive data’ which cannot provide sufficient detailed to be further interpreted • Qualitative studies on dementia
Study types	• Peer-reviewed research articles	• Review articles, books, conference papers, theses
Time frame	• 2011 to 2022	• Before 2011
Language	• English	• All other languages
Population	• Family caregivers who are current caregivers or had the experience of caring for adults over the age of 50, or who are “older/elderly” if chronological age is not specified • Caregivers with an immigrant background from Asia, Africa and South America residing in Europe	• Family caregivers who do not have experience of caring for adults over the age of 50, or who are “older/elderly” if chronological age is not specified • Caregivers who do not have an immigrant background from Asia, Africa and South America
Study country (setting)	• European countries	• Countries outside Europe
Phenomenon of interest	• Studies explicit about family caregivers’ experiences of caregiving to older adults in the family	• Studies where the primary interest is the experiences of the care receivers or health personnel

**Table 3:** Quality assessment of primary studies using CASP checklist

	Are the results valid?						What are the results?			Will the results help locally?
Article ref	1. Aim	2. Method	3. Design	4. Recruitment	5. Data collection	6. Relationship	7. Ethics	8. Data analysis	9. Findings	10. Valuable
Arora et al_2019	Y	Y	Y	Y	Y	Y	Y	Y	Y	Y
Arora et al_2020	Y	Y	Y	Y	Y	Y	Y	Y	Y	Y
Cowan_2014	Y	Y	Y	Y	Y	CT	Y	Y	Y	Y
De Tavernier & Draulans_2018	Y	Y	Y	Y	Y	N	CT	Y	Y	Y
Giunntoli & Cattan_2012	Y	Y	Y	Y	Y	Y	Y	Y	Y	Y
Greenwood et al_2016	Y	Y	Y	Y	Y	CT	Y	Y	Y	Y
Ismail _2021	Y	Y	Y	Y	Y	CT	N	CT	Y	Y
Nielsen et al_2018	Y	Y	Y	Y	Y	Y	Y	Y	Y	Y
Parveen et al_2011	Y	Y	Y	Y	Y	Y	Y	Y	Y	Y
Pound & Greenwood_2016	Y	Y	Y	Y	Y	N	Y	Y	Y	Y
Van Eechoud et al_2016	Y	Y	Y	Y	Y	Y	Y	Y	Y	Y

*Y=* Yes, *N* =No, *CT* =Cannot tell

### Phase 3: reading the studies

The selected studies, comprising two from Norway [41, 42], two from Denmark [43, 44], two from Belgium [45, 46] and five from the UK [47–51] were read repeatedly to aid familiarization with the concepts and metaphors of these studies in detail [32, 34]. There were variations in caregivers’ experiences based on the health conditions of the older adults. Those who were caring for older adults without a particular diagnosis of disease shared more experiences related to providing practical assistance [41, 43, 44, 46, 51]. In contrast, caregivers of older adults with cancer [45], stroke [48–50] and terminal illness necessitating palliative care [47] shared more about their perspectives on health and social care services in addition to practical assistance. One of the included studies was a single-case study of a family, with rich details about the children’s caregiving experiences with their mother [44]. Another study was a case study of a Turkish community in the town of Genk, Belgium [46]. Though the two included studies from Norway [41, 42] reported findings from the same group of participants, their data collection methods, themes and quotes differed.

The first author extracted characteristics of these primary studies, including authors, titles, study aims, country of study, health condition of older adults, methods and participants to provide the context for interpretations and explanation of each article (Table 4). The informal caregivers in the included studies were predominantly children (daughters, daughters-in-law, sons and grandchildren) and a few were spouses of care receivers. Most of the caregivers were female. Once the 11 articles were studied in detail, the first order constructs (participants’ quotations) and second order constructs i.e., primary authors’ interpretations (metaphorical themes and concepts) [34] were extracted from these studies by coding concepts in the qualitative analysis software package NVivo 12 [52].

**Table 4:** Study characteristics of the included studies

Author/year published	Title	Aim of the study	Older adults’ health status	Country	Study design	Data Collection	Data Analysis	Participants’ immigrant background	Participants (inclusive in our study)
Arora et al., 2019	Ethnic boundary-making in health care: Experiences of older Pakistani immigrant women in Norway	To explore how older Pakistani women experience their healthcare interactions in Norway	Generacaregiving	Norway	Qualitative study	Semi-structured interviews, Focus groups	Thematic Analysis (Braun & Clarke, 2006)	Pakistani	10 informal caregivers (all female)
Arora et al., 2020	Female Pakistani carers’ views on future formal and informal care for their older relatives in Norway	To explore female Pakistani carers’ views on the future formal and informal care and healthcare accessibility of their older relatives in Norway	Genera caregiving	Norway	Qualitative study	Semi-structured interviews, Field notes	Thematic Analysis (Braun & Clarke, 2006)	Pakistani	10 informal caregivers (all female)
Cowan, 2014	The lived experiences of the Sikh population of southeast England when caring for a dying relative at home	To explore and understand the Sikh population of southeast England’s experiences of care for a dying relative at home without support from local SPC team	Palliative care	UK	Phenomenological study	Semi-structured interviews	Interpretative Phenomenological analysis (Smith et. al, 2009)	South Asian (Punjabi-Sikh)	5 informal caregivers (all female)
De Tavernier & Draulans, 2018	Negotiating informal elder care, migration and exclusion: the case of a Turkish immigrant community in Belgium	To go beyond the general discussion on gender inequalities and care and argue that exclusion is a core mechanism through which informal care can be organized or even guaranteed	Genera caregiving	Belgium	Qualitative case study	Semi-structured interviews	Intersectional approach (Crenshaw 1989)	Turkish	6 informal caregivers (all female)
Giuntoli & Cattan, 2012	The experiences and expectations of care and support among older migrants in the UK	To investigate the accessibility and acceptability of care and supported services in Bradford, UK, a city with a large migrant population	Genera caregiving	UK	Qualitative study	In-depth interviews, Focus groups	Framework-Analysis (Ritchie & Lewis, 2003)	Pakistani, Indian, Bangladeshi, Afro- Caribbean	33 caregivers (didn’t differentiate in numbers between BME group and other European group)
Greenwood et al., 2016	Qualitative focus group study investigating experiences of accessing and engageing with social care services: perspectives of carers from diverse ethnic groups caring for stroke survivors	To explore BME and white British carers’ experiences of accessing and receiving social care services in the community, focusing on similarities and differences between ethnic groups	Stroke Survivors	UK	Qualitative study	Focus groups	Thematic analysis	Pakistani, Indian, Black African, Black Caribbean	31 informal caregivers
Ismail,2021	Care in practice: negotiations regarding care for the elderly in multigenerational Arab Muslim families in Denmark	To explore how caring for elderly people with health problems at home raises specific questions about obligations and triggers negotiations across genders and generations	Genera caregiving	Denmark	A single case study	Formal and informal interviews, Field observation	_	Arab Muslim	5 informal caregivers (4 female, 1 male)
Nielsen et al., 2018	“Caught in a Generation Gap”: A Generation Perspective on Refugees Getting Old in Denmark—A Qualitative Study	To gain better understanding and insight into the care needs of refugee families with aged relatives who are vulnerable in a resettlement country	Genera caregiving	Denmark	Hermeneutic phenomenological approach	Semi structured interviews, Focus group interviews	Systematic text condensation (Malterud, 2012)	Iraqi, Lebanese, Palestinian, Somali	22 informal caregivers (15 female, 7 male)
Parveen et al., 2011	Ethnic variations in the caregiver role: A qualitative study	To explore the experience of British South-Asians in comparison with White-British caregivers, in order to address gaps in the current caregiver literature	Cancer, Stroke & others	UK	Mixed-methods study	Semi-structured interviews, Focus group	Thematic content analysis (Braun & Clarke, 2006)	Pakistani, Indian, Bangladeshi	21 informal caregivers (19 female and 2 male)
Pound & Greenwood, 2016	The human dimensions of post-stroke homecare: experiences of older migrants in the UK	To explore the post-stroke experiences of older carers from BME and White British populations receiving homecare	Stroke Survivors	UK		Semi-structured interviews	Thematic Analysis (Braun & Clarke, 2006)	Pakistani, Indian, BlackAfrican, Black Carribean	38 informal caregivers (28 female, 10 male)
Van Eechoud et al., 2016	Caring for Family Members Older Than 50 Years of Turkish and Northwest African Descent: The Meaning of Caregiving	To gain insight into the experiences and perceptions of families with Turkish or Northwest African backgrounds, caring for cancer patients aged over 50	Cancer	Belgium	Constructivist Grounded theory	Loosely structured interviews	Qualitative Analysis Guide of Leuven (Dierckx et al., 2012)	Turkish, Northwest Africa	32 family caregivers (22 female, 10 male)

### Phase 4: determining how the studies are related

Determining how studies are related involved creating ‘a list of key metaphors or concepts’ from the primary studies [32]. The studies were related due to their similar purpose of exploring the experiences of informal caregivers. NVivo helped keep track of the coding and conceptual development while being able to return to the primary studies [53]. Examples of a few metaphorical themes were “moral meaning of caregiving”, “inaccessibility to care and support services” and “negotiation in caregiving”.

Two studies from Belgium [45] and the UK [47] with the highest scores in the CASP appraisal (Table 3) were used as index studies for commencing coding in NVivo. This process helped translate concepts from these two studies to other studies and, therefore, shaped the analysis [34]. Later, other studies were coded gradually. It resulted in common and recurring concepts (similar code), as well as additions to the previous coding (new code) which were further clustered into different categories. As a result, we obtained eight descriptive categories in this phase: 1) Ideal perceptions of continuing caregiving to older family members; 2) Practical concerns of continuing caregiving; 3) Fear of social exclusion and inaccessibility of appropriate formal services; 4) Determining factors for taking on a caregiving role; 5) Perceived need of sharing caregiving responsibilities; 6) Changing caregiving approaches; 7) Consequences of caregiving; and 8) Coping strategies as an individual responsibility.

### Phase 5: translating the studies into one another

This phase involved translating the studies into one another, which required comparing the metaphors and concepts (themes) from the individual studies with each other [32]. First, we started by summarizing one of the index studies from Belgium [45], which resulted in an overall theme of “moral and practical meaning of caregiving”. Second, we looked for this theme in another index study, which was conducted in the UK [47], and found this theme to be common to both studies. Third, other themes such as gender as a determining factor in caregiving, and coping strategies, were added. The metaphors and concepts of the included studies could be grouped around two overarching themes. First, why they care; and second, how they care. All 11 studies were compared, and they were covered by the eight descriptive categories mentioned before. During this process, these eight descriptive categories were further refined.

### Phase 6: synthesizing translation

This phase is described as ‘making the whole into something more than the parts alone imply’ [32], which can be divided into two stages; reciprocal synthesis and line of argument synthesis. The stage of reciprocal synthesis involves deciding whether the studies are sufficiently similar in their focus for establishing relationships to allow for a reciprocal translation synthesis [34]. We summarized the shared themes across the primary studies by comparing the first order constructs (metaphors) and second order constructs (concepts). This iterative process continued until we developed the four main themes. The Table 5 provides an overview of reciprocal synthesis of translations.

**Table 5:** Reciprocal synthesis of translations

Descriptive Categories	Main Themes
• Ideal perceptions of continuing caregiving to older family members • Practical concerns of continuing caregiving • Fear of social exclusion and inaccessibility of appropriate formal services • Determining factors for taking on a caregiving role • Perceived need of sharing caregiving responsibilities • Changing caregiving approaches • Consequences of caregiving • Coping strategies as an individual business	• Strong care norms towards parents • The moral dilemma of continuing care • Uneven care sharing • Use of formal care services

The next stage is a line of argument synthesis, meaning that there is an “interpretation of the relationship between the themes, which further emphasizes a key concept that may be hidden within individual studies in order to discover the whole from a set of parts” [32]. This is classified as a further higher level of interpretation synthesis which provides scope for developing new insights [34] or as the new ‘storyline’ or overarching explanation of a phenomenon [33]. The main four themes we obtained through reciprocal synthesis were further synthesized to form a line of argument. As such, we developed one overarching core theme, “changing dynamics of caregiving”.

### Phase 7: expressing the synthesis

The synthesis is presented in the form of a written report targeting the audiences (researchers, academics, health professionals and policymakers) of this article.

## Results

The core theme that emerged through our analysis was “changing dynamics of caregiving”. The four themes, representing informal caregivers’ experiences of caring for older family members with an immigrant background in Europe – namely strong care norms towards parents, the moral dilemma of continuing care, uneven care sharing, and use of formal care services – are presented in the following sections, with illustrative quotations from the primary studies.

### Strong care norms towards parents

Strong care norms i.e., the perceived responsibility of caring for family members, was high among several caregivers across many of the studies [41, 43–46, 48, 49]. Some participants illustrated the strong care norms of children towards their parents. They acknowledged that their willingness to continue providing care to their parents was related to cultural and religious obligation [45, 46, 48] and expressed it as a matter of respect for their respective cultures [43, 46]. One female medical doctor and a caregiver of Turkish origin said, *“It is partly inspired by culture, partly by religion, and our faith dictates that we must continue to look after our parents until the end.”* [46].

Some expressed that they feel intrinsically motivated to continue caregiving as that was the right thing to do [45, 47] and expressed pride and honor in caring for their parents [41, 43, 48]. In a study conducted in Belgium among caregivers of Turkish and Northwest African descent, a family (including son, daughter, and wife) stated that admitting their father/husband to a palliative care unit went against their principles regarding fulfillment of the moral obligation of caregiving. The son recounted, *“…If you have children, your children need to take care of you in bad times. And if you don’t do that, it is bad also. As to say, it is a bad example for society too. (…)”* [45]. The feeling of satisfaction for fulfilling cultural, religious, and moral duties was common among many caregivers [41, 43–45, 47–49] and constituted a motivation to continue their caregiving responsibilities.

### The moral dilemma of continuing care

The ideal perceptions described in the previous theme were often in conflict with the moral dilemmas of continuing caregiving. For the first generation, their image of eldercare was based on their experiences in their country of origin before they migrated to Europe. As a result, the transmission of care norms to the younger generation was based on the first generation’s socialization dating back several decades [41, 44, 46]. Many of the older family members preferred to be cared for by their children than others [41, 44, 46–48], and not adhering to the preferences of older family members was perceived to weaken the central aspects of filial piety [43, 44].

Many Pakistanis and Turkish participants from studies in Norway [41] and Belgium [46] respectively anticipated the concern about what other people might say about choosing alternative forms of care [41, 46]. One participant with Turkish background in Belgium noted that resisting or failing to fulfill the community’s expectations would bring feelings of shame, guilt, and social exclusion. She said, ‘*… when you have a parent in a home* (care home)*, the community treats you like an outcast…So it is still somewhat of a taboo to place your parents in an old people’s home’* [46].

Many participants stated that they felt they had no choice but to adopt the role of the caregiver given the lack of alternatives [43, 45, 47, 48]. One 34-year-old female participant with Northwest African immigrant background described how choosing alternatives to informal caregiving was perceived as an immoral practice in their culture, meaning that children are not grateful to their parents’ efforts in raising them [2]. Another participant from a study in Norway noted how adult children’s jobs and household responsibilities are not considered a sufficient reason to justify choosing an alternative to informal caregiving [41].

A few caregiver participants, who migrated to Europe with their parents in early childhood, asserted that they will be less dependent on their children in their old age [45, 47], and said the younger generation (who were born and raised in Europe) may not be so dedicated to the caring norms espoused by older family members [47]. The younger generations’ perceptions of caregiving were found to be less influenced by the older adults’ definition of ideal caregiving, and they described it as a sense of duty [41, 44, 49]. One participant aged 23 with Palestinian background and living in Denmark who contributes (along with her father and other siblings) to the care of her grandmother said, “*She (grandmother) says: are you here to take care of me or to do your own stuff? Unfortunately, my cousins have accustomed her to ask them to do whatever she wants them to do. To be honest, I do not enjoy her company, but I feel like it is my duty”* [44]. As such, younger generations were found to have more leeway from the home country’s caregiving perspective and therefore had more flexibility in choosing alternatives to providing informal care [41, 44, 45].

### Uneven care sharing

Family caregivers were responsible for providing practical assistance to older adults and helping them navigate the health system [41–45, 47]. Some participants helped with household tasks like cleaning, laundry, shopping, preparing food, and personal hygiene [41, 43–45]; others acted as mediators between the health system and their older family members, and often made appointments and helped with the transportation to hospitals [43, 45, 47, 49, 50]. The roles were not only divided differently but sometimes also unevenly among the family members.

Gender was one of the most prominent factors in determining the caregiver role [41–50], with some women feeling obliged to take on a caregiving role [41, 46, 49]. Most caregiving tasks revolved around household chores [41, 46] and daughters were normatively expected to take these responsibilities [46]. A female caregiver of Turkish origin, looking after her mother in Belgium, said, *‘I was always expected to clean my parents’ home, from top to bottom… All of it used to be my job… But I think that my brothers would have struggled with looking after my mum, that is true’* [46].

Many caregivers discussed the heavy workload attached to caregiving [41, 43, 44, 46], where they hardly got time for themselves or other family members [47, 48]. Some complained about limited mobility and felt restricted in doing things or going places with their children. Some participants talked about the adverse health effects resulting from the constant pressure of caregiving responsibilities [43, 46]. Many female caregivers faced great role conflict in their attempts to juggle occupational demands, childrearing, and the demands of caring for older relatives [41, 43, 46, 48].

Many of them emphasized the burden of being a sole caregiver and expressed preferences for sharing the care burden with siblings and their children [43, 44]. The lack of extended family or living in proximity to the care recipient limits the possibility of sharing caregiving responsibilities [41, 45]. A few participants who get support from other family members noted that their experience would not have been possible without family support and teamwork [47]. A caregiver with Punjabi background who takes care of her mother-in-law in England expressed teamwork as a factor in managing to fulfill care at home, *‘I explained to the family that I needed to go to work from 10am to 3 pm and when I come home, I take over. I had the help, the whole family helped’* [47].

On the contrary, some participants got either too little help, or no help at all from their other family members [43, 46–48]. They received help either for instrumental care tasks such as attending doctor appointments or personal hygiene tasks. It made some caregivers angry, demotivated to provide care, and created conflicts among siblings [44–48]. A daughter with Moroccan background who cared for her mother in Belgium reported that wealthier siblings would have more power to avoid their caregiving responsibilities [45]. Both the studies conducted in Belgium showed that the unemployed or those on low wages were expected to take care of duties [45, 46]. A few participants also noted that living near to their parents’ house [41] and having the skills to navigate the health system [45] also contributed to them becoming caregivers.

In many cases, caregivers were left with being sole caregivers and bearing caregiving challenges alone. Only a few studies briefly mentioned how caregivers cope with the caregiving challenges [44, 47, 48]. Some avoid conflicts with care receivers [44] and siblings [44, 46] to avoid further stress from being the only caregiver. A few caregivers with South Asian backgrounds revealed that they used to cry when caregiving became too much for them and felt hopeless [47]. At the same time, a few of them also relied on religion for coping [47, 48], as they found the strength to continue their caregiving [47] and to gain spiritual blessing for their caregiving role [48]. Some of the participants noted that they think less about the burden of caregiving. They reported that the burden becomes habitual eventually for lack of other alternatives, and they try to suppress negative aspects, thinking that it will only increase their stress [48]. A British-Bangladeshi female of aged 40 who was caring for her husband in the UK expressed*, ‘Even if it’s a burden, you have to do it. If there’s no one else in the family, how else is it going to get done?* [48].

Regardless of mutual acceptance or conflict among siblings for sharing caregiving responsibilities, some studies emphasized changing caregiving approaches [44, 46, 48]. Older family members preferred co-residence with their family members [41, 43, 44]. However, new approaches such as children staying nearby to provide care, rotational caregiving whereby parents live one week with each of their children, or children visiting the older adults each week were emerging, as shown by a study among Arab Muslim families in Denmark [44]. In one study from Norway, a few participants discussed the necessity of searching for home care services regardless of their parents’ reservations, when they no longer would be able to take care of them [41].

### Use of formal care services

Using home care services for older family members was rare in contexts with no specific disease diagnosis [43, 47, 49–51]. Our analysis of the primary studies demonstrated that many participants had negative views toward social and health services [41–43, 47–51]. Others had a fear that outsiders would not provide the same level of care as family members [41, 48], and preferred for their older relatives to die at home surrounded by relatives, as shown by a study among South Asian participants in the UK [47]. Pound & Greenwood (2014) described the participants’ contact with the health system as stressful since it failed to address the emotional aspects of caregiving and their concerns for their older family members [50].

Many participants identified language [42, 43, 46, 49, 50] and limited awareness of the available eldercare policies, benefits, and services [46, 47, 50] as significant barriers to accessing health services, which in turn increased older adults’ dependency on their informal caregivers [42, 43, 46]. Within the context of accessing care services, Greenwood et al. emphasized the difficulty of caregivers with unfamiliar terminology, unresponsive services, and heavy paperwork making services even more inaccessible [49]. Though limited language proficiency was found as the main barrier to accessing health services, many participants believed that judgmental attitudes toward immigrants went beyond the language barrier [41, 42, 46, 48, 49]. Some needed to exaggerate their health problems to be heard [42] and fight the system to get services in time [49, 50]. As such, family caregivers feel unsupported by health professionals within home care services [47, 49, 50].

The time constraint of health professionals providing home care services was one of the significant issues reported by caregivers who are caring for older adults after discharge from hospital [49–51], followed by delayed provision of services [50]. Some participants showed concern for the lack of cultural sensitivity within health services [42, 48] and a few caregivers mentioned that revealing their intimate bodies to strangers was concerned with disrespect, neglect, and injustice in their cultures [44, 51].

## A line of argument synthesis

**Fig. 2:**
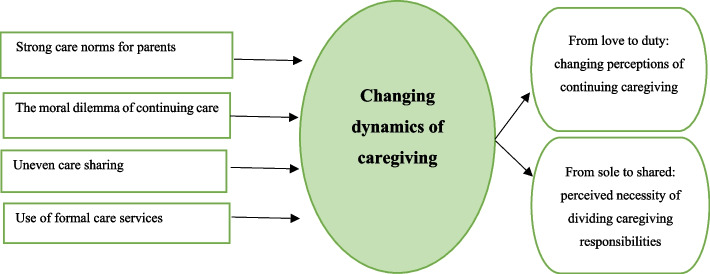
A line of argument synthesis of ‘changing dynamics of caregiving’

Though expectations of caring for older relatives were high within families of immigrant background, our results show that the dynamics of caregiving are changing, as illustrated in Fig. 2. It signifies that the meaning and approaches attached to caregiving are constantly changing and so are the experiences of family caregivers. The perceptions of continuing caregiving for older family members varied from satisfaction of cultural fulfillment to burdening duty, whereas the perceived necessity of dividing caregiving responsibilities among family members is growing, given their circumstances in the host countries.

## Discussion

In this review study, we performed a meta-ethnographic synthesis exploring immigrants’ caregiving experiences with older family members from Asia, Africa and South America living in Europe. Our main findings illustrate the meaning of caregiving across the different generations caring for older family members with an immigrant background and show how caregiving to older adults varies in the given context of caregivers.

The review study highlighted cultural norms, filial obligations, reciprocity, and moral obligations as ideal reasonings for continuing care. This is in line with another previous study that shows how relational, normative, moral and affective dimensions of caregiving reflect notions of care [19]. The care norms among caregivers were strong and were a part of cultural and religious obligations. Providing care was perceived as an opportunity to reciprocate parental love and support by adult children [2, 18]. Similarly, the studies in our review study showed that many caregivers felt gratitude toward their parents and were intrinsically motivated to provide care as they considered it was the right thing to do. Other review studies reported that immigrant-background caregivers have a deep sense of pride and satisfaction in caring for a family member [12, 13], leading to psychological rewards and personal growth [25, 26].

Nevertheless, there appears to be an intergenerational shift in caregiving motivation for some, from a culturally normative obligation to a morally induced filial duty/familial responsibility. Although the motivation might change for some, for others it is either mixed or unchanged as the feeling of satisfaction for fulfilling cultural, religious, and moral duties was common among many caregivers. Many caregiver participants who were born in their parents’ home country but migrated with their parents in early childhood, felt obliged to care and adhere to caregiving norms. However, they expressed having fewer expectations to be cared for by their family, as shown by a review study on older immigrants in the United Kingdom [23]. Immigrants and their children’s cultural traditions contain two or more competing and sometimes contradictory cultural and moral systems that are associated with different places [24]. Younger generations who have lived in the receiving country for a more extended period seem more likely to internalize natives’ values, norms and expectations about family relationships [2]. As such, an acculturation gap arises between the younger generation and their parents, because of this dual socialization to their parents’ values as well as to the values of the destination society [54].

Our review highlighted the caregiver participants’ desire to adjust traditional caregiving norms in line with the host country’s caregiving perspectives, so that they could have a choice and alternatives to care for their family members. However, our review also shows that when providing caregiving to older family members is the norm, unwillingness or failure to comply with such norms leads to guilt, shame, and social exclusion among caregivers. This finding is also supported by an empirical study conducted in Norway which showed that seeking help from healthcare services could hence be considered a sign of failure to fulfill one’s familial obligations [17]. As such, the caregivers experience a moral dilemma between their changing values due to acculturation and the traditional expectations of older community members.

Though some caregivers wished to continue caregiving and felt morally obliged to do so, adhering to the ideal caregiving roles was difficult as their caregiving role often overlapped with childrearing. This notion is often addressed as sandwich caregiving [55]. Further, another qualitative study highlighted the intra-role conflict experienced by caregivers due to conflicting expectations from their different roles, eldercare, childrearing, and needs of family and friends, making caregiving a burden for them [56]. Thus, there is uncertainty about their ability and willingness to assume full responsibility for such care [57]. Consistent with previous literature, our review highlighted the intra-role conflict of caregivers and conflicting expectations. Such situations can lead to caregivers experiencing moral dilemmas resulting in complex decision-making between the continuation of informal caregiving or choosing alternatives to such caregiving.

The participants in the included studies in this review were primary caregivers, and most of them shared the role of caregiving, albeit unevenly. It resulted in higher caregiving responsibilities among family members, particularly for women. Our review showed the notable gender disparities in the distribution of caregiving duties, where women predominantly took on the caregiving role. Similar findings are also present in previous literature [2, 9, 12, 13, 20]. Most caregivers were daughters, daughters-in-law, or wives to care receivers. In general, daughters, on average, provide twice as much care to their ageing parents as sons [58]. This disproportionate involvement of women in caregiving is often attributed to gender stereotypes that frame caregiving as a “feminine type” of activity [21] and results in women anticipating their caregiving role not as something new but rather as an extension of their existing feminine roles [20]. Further, who takes up the caregiving role and to what extent they experience challenges is influenced by their specific circumstances of family support and relationships, income, employment, and proximity to the care recipient, among others. In line with this finding, another study has argued that social circumstances may allow only certain people to cope effectively [19]. Intertwined with gendered expectations of caregiving, women are likely to experience additional challenges in navigating their role as a caregiver [16, 19, 20]. A systematic review on gendered experiences of informal caregiving for older people demonstrated a higher burden of caregiving among women than men, stating that women are emotionally involved in caregiving and less likely to ask for support. In contrast, men are task-oriented and more likely to ask for support [20].

Most of the included studies in the review reported the high care burden among caregivers resulting in burnout, characterized by constant stress, limited mobility, and lack of time for oneself. Several previous studies also emphasized the physical, emotional, psychological, and socio-economic burden experienced by caregivers [12, 13, 16, 28] and showed them having a higher risk of adverse health consequences, such as depression, with higher subjective burden of care [27]. A noticeable finding in our review is the difficulty, particularly for sole caregivers, to continue their caregiving and highlighted their desire to share caregiving responsibilities with other family members. As a result, rotation care practices were evolving, either by having older adults live with different family members or by having different family members take turns to move in with older adults, visiting weekly or staying nearby. There was a shift of caregiving to older adults from individual to group responsibilities, and caregivers expressed caregiving as teamwork. A study from Denmark among minority families demonstrated similar results, concluding that new caregiving approaches emerged as ways to alleviate or distribute the burden of care between siblings [18]. Other studies [12, 13, 59] mentioned alternative caregiving approaches like employing a full-time maid or undocumented domestic helper, temporary relocation to the home country, or organizing a care marriage.

Furthermore, our review showed that caregivers’ desire to share caregiving responsibilities with family members often caused family conflicts. Similarly, a qualitative study from Netherlands among family caregivers with immigrant backgrounds reported disappointments among caregivers over non-caregiving family members, which led to tensions and sometimes conflicts, usually left unspoken, that later contributed to their emotional and physical exhaustion [16]. Hence, the sole caregivers are left vulnerable with a higher burden of care. Regardless of the high caregiving burden, caregivers’ ways of coping were found to be limited in our study. They often released their stress by crying, sought religious support by praying or tried to ignore their burden until those stresses become habitual to them. The gender stereotypes which make caregiving a normative part of women’s family life may impact the choices and coping strategies that eventually prohibit women from seeking help and interventions [20]. Passive coping strategies like forgiveness, tolerance, or contentment were found among Chinese American caregivers [60]. Though supports such as training, respite care, and counseling have effectively reduced the burden of caregiving [9], such support/coping strategies were lacking among participants in our review study, demonstrating the inaccessibility of culturally sensitive healthcare services among family caregivers.

Many studies highlighted language as a significant barrier to accessing available services [1, 12–14, 61], but our review asserts that the inaccessibility of available services goes beyond some immigrants’ limited language skills. The lack of information about available formal services further makes these services inaccessible to those who want to explore alternatives to informal caregiving, hence limiting them from seeking support. Similarly, the systematic review about informal caregiving among migrants mentioned that the preconceived ideas about the inappropriateness of support and public healthcare might also prevent caregivers from seeking or accepting formal support. Moreover, two systematic reviews [12, 13] on family caregivers’ experiences of providing care for family members from minority ethnic groups living with dementia reported poor health literacy among caregivers.

In accordance with our review, previous research [2, 14, 16–18, 61] show that the caregiving norm and the perceived responsibility of ‘taking care of their own’ hinder the use of home-care services and other forms of professional care among people with an immigrant background. The perceived poor cultural sensitivity of available services makes caregivers reluctant to search for alternatives to informal caregiving. Many caregivers who had experience of using formal services in our review had negative experiences with health care professionals. They had skeptical views about health services/care institutions and felt ignored by the health system, which was not uncommon among family caregivers of the other two review studies on dementia [12, 13]. The current review showed caregivers’ stressful experiences with the health system and mentioned that it often failed to address the emotional aspects or what caregiving to their older family members means to them. Some studies also reveal that a lack of cultural competency among professionals’ results in poor acknowledgment of older adults’ religious rituals, dietary practices, or gender matching [1, 14, 61]. This shows the need for cultural competence training for health and long-term care professionals. Support for informal caregivers would help avoid overburden and ensure access to formal care when needed.

Our meta-ethnographic review contributes to a better understanding of informal caregiving and has critical implications for further research. Knowledge about differing meanings of caregiving and caregiving responsibilities enhances understanding about informal caregivers’ situations and would aid in reducing their care burden while ensuring access to formal care when needed. There is also need for more knowledge about how the changing dynamics of caregiving may be further influenced by the institutional settings, immigrants’ ethnic backgrounds, and other social circumstances. Further, it will be useful to explore the care expectations across different generations, particularly the children of immigrants, and to see how their socialization in two cultures influences care norms as they age. Further, more knowledge about caregivers’ coping strategies, and their motivations for adopting them, would aid in developing better support measures in the long term.

### Implications for practice

The current review study points toward many implications for clinical practice. As poor information about the available health and social services was a significant barrier to accessing health among ethnic minorities, these communities must be reached with information to help them navigate the health system. They could be reached by coordinating with key persons, religious leaders, or women’s organizations for awareness programs.

Although the care burden was high among family caregivers, the option to access nursing homes and other formal healthcare services contradicted their strong care norms, represented by cultural and moral obligations. Family caregivers’ needs and support should be prioritized, and care burdens must be shared to complement their perceived care norms and reduce their care responsibilities. A home-based care system with better coordination between health personnel and family members could be an alternative if the understanding of ‘appropriate care’ is mutual.

Those searching for alternatives should be met with appropriate health and care services in terms of language, culture, religion, and lifestyle, delivered in a non-judgmental way. In case of limited language skills, offering support from a professional interpreter is critical, or professionals with bilingual skills could help provide quality care.

### Strengths and limitations of the study

Among the several strengths of this review is the use of systematic methods and multiple databases to identify relevant studies. All authors participated in screening, and each eligible study was screened by two authors independently to reduce potential bias. Further, we attempted to present the analysis process rigorously to obtain transparency in the meta-ethnographic synthesis process. Though we have already justified the inclusion criteria of our review study, expanding the geographic scope beyond Europe could add to the richness of data from different contexts. Another potential limitation is that the study included only peer-reviewed articles in the English language published from 2011–2022, which might have excluded possibly relevant studies which do not fit into these categories. Most participants in the included studies were female. Therefore, while talking about gendered caregiving responsibilities, it predominately represents the caregiving experiences of females. Though male family caregivers were few in number, further exploring men’s experiences could shed more light on the gendered aspects of informal caregiving.

## Conclusion

Strong care norms towards caring for older family members is a reality for many immigrant communities. The motivation behind caregiving is rooted not only in culturally normative obligations but also in morally induced familial responsibilities. The motivation and approaches to caregiving, while unique to individual caregivers, are also dynamic. Family caregivers’ contextual realities pose constraints on the continued expectations of informal care. Caregiving is thus burdensome, especially for those without formal and informal support systems. This is compounded by the continued gendered nature of caregiving which often results in greater expectations and care burden for women.

## Supplementary Information

**Additional file 1.**

**Additional file 2.**

## Data Availability

All data generated or analyzed during this study are included in this published article [and its supplementary information files].
