# Supplementary material for: Changing dynamics of caregiving: a meta-ethnography study of informal caregivers’ experiences with older immigrant family members in Europe
Source: BMC Health Serv Res. 2023 Jan 17;23:43. doi: 10.1186/s12913-023-09023-4 (PMC9847080; doi:10.1186/s12913-023-09023-4)
Supplement: Supplementary file 2 — Additional file 2. [file 12913_2023_9023_MOESM2_ESM.docx]

| UNIVERSITETSBIBLIOTEKET, RESSURSGRUPPE FOR LITTERATURSØK |
| --- |
|  |
| **Literature search documentation** |
| **Project:** |
| Update search - Immigrants’ caregiving experiences to older family members |
| **Date:** |
| 30.06.2022 |

# **Summary of the search**

| **Type of search:** | Update - Systematic literature search.  *NB Duplicate references for 2021 included in initial search are not removed.* |
| --- | --- |
| **Databases:** | Medline Ovid, Embase Ovid, PsycInfo Ovid, SocIndex EBSCOhost, Cinahl EBSCOhost, Scopus, Social Care Online, ASSIA proquest and Google Scholar |
| **Population:** | Immigrants as caregivers to older family members |
| **Outcome:** | Experiences |
| **Year, limitation:** | 2021-2022 |
| **Language, limitation:** | No limit |
| **Study design:** | Qualitative research (both subject terms and text words) |

**Results**

| ***Databases*** | ***Results*** |
| --- | --- |
| Medline via Ovid | 249 |
| Embase via Ovid | 329 |
| PsycInfo via Ovid | 132 |
| Cinahl via EbscoHost | 165 |
| SocIndex via EbscoHost | 92 |
| Scopus | 250 |
| Social Care Online | 25 |
| ASSIA proquest | 75 |
| Google Scholar | 150 |
| *Total including duplicates* | *1467* |
| *Total after duplicate removal* | *741* |

# **Search history**

**Database:** Ovid MEDLINE(R) and Epub Ahead of Print, In-Process, In-Data-Review & Other Non-Indexed Citations and Daily 1946 to June 27, 2022

**Date:**  28.06.2022

**Results:** 249

| **#** | **Searches** | **Results** |
| --- | --- | --- |
| 1 | "Aged, 80 and over"/ or Aged/ or Frail Elderly/ or Geriatrics/ or Dementia/ | 3445277 |
| 2 | ((aged or old*) adj2 (people or women or person* or men or immigrant* or minorit* or parent*)).tw,kw,kf. | 205233 |
| 3 | (senior or seniors or geriatric or elder or elders or elderly or dement* or aging or ageing or old age).tw,kw,kf. | 723747 |
| 4 | old*.ti,kw,kf. | 253829 |
| 5 | or/1-4 | 3973992 |
| 6 | Caregivers/ or Caregiver burden/ | 46269 |
| 7 | (caregiver* or care giver* or caretaker* or care taker* or carer*).tw,kw,kf. | 103556 |
| 8 | ((children* or son or sons or daughter* or offspring* or sibling* or brother* or sister* or wife* or wives or husband* or partner* or spous* or married* or famil* or parent* or father* or mother* or next of kin* or kinship* or significant other* or relative or relatives or informal or unpaid or old* or elder* or aged or aging or ageing or senior or seniors or geriatric) adj3 (care or caring or caregiving)).tw,kw,kf. | 107481 |
| 9 | Adult Children/ or Siblings/ or Spouses/ or Family/ or Nuclear family/ or Family relations/ or parents/ or fathers/ or mothers/ | 242165 |
| 10 | (caring or caregiving or (care adj1 giving)).ab. | 51001 |
| 11 | 9 and 10 | 8037 |
| 12 | (caring or caregiving or (care adj1 giving)).ti,kw,kf. | 18581 |
| 13 | 6 or 7 or 8 or 11 or 12 | 214047 |
| 14 | "Emigrants and Immigrants"/ or Undocumented Immigrants/ | 14874 |
| 15 | Ethnic Groups/ | 69635 |
| 16 | Minority groups/ | 16681 |
| 17 | Refugee/ | 12359 |
| 18 | cross-cultural comparison/ | 27158 |
| 19 | cultural characteristics/ | 16844 |
| 20 | cultural diversity/ | 12606 |
| 21 | "Emigration and Immigration"/ | 26011 |
| 22 | (Immigrant* or migrant* or multicultur* or intercultur* or minorit* or ethnic* or multiethnic* or racial* or refugee* or non western* or nonwestern* or asylum seeker*).tw,kw,kf. | 314087 |
| 23 | or/14-22 | 410450 |
| 24 | qualitative research/ | 74961 |
| 25 | Focus Groups/ | 34440 |
| 26 | Interview/ | 30340 |
| 27 | narration/ | 9677 |
| 28 | Grounded Theory/ or Hermeneutics/ | 2945 |
| 29 | Attitude/ | 51943 |
| 30 | Behavior/ | 30162 |
| 31 | Perception/ | 41562 |
| 32 | (Qualitative or Narrative or Phenomenolog* or Hermeneutic* or interview* or Grounded theor* or Ethnograph* or themes or Attitude* or Behavior* or behaviour* or Perception* or View* or experience* or focus group*).tw,kw,kf. | 3727324 |
| 33 | or/24-32 | 3780381 |
| 34 | 5 and 13 and 23 and 33 | 2167 |
| 35 | limit 34 to yr="2021 -Current" | 249 |

**Database:** Ovid- Embase 1974 to 2022 Week 25

**Date:**  28.06.2022

**Results:** 329

| **#** | **Searches** | **Results** |
| --- | --- | --- |
| 1 | aged/ or frail elderly/ or very elderly/ or geriatrics/ or dementia/ | 3478263 |
| 2 | ((aged or old*) adj2 (people or women or person* or men or immigrant* or minorit* or parent*)).tw,kw,kf. | 267087 |
| 3 | (senior or seniors or geriatric or elder or elders or elderly or dement* or aging or ageing or old age).tw,kw,kf. | 981874 |
| 4 | old*.ti,kw,kf. | 297708 |
| 5 | or/1-4 | 4144929 |
| 6 | Caregiver/ or Caregiver burden/ or Caregiver burnout/ or Caregiver support/ | 108907 |
| 7 | (caregiver* or care giver* or caretaker* or care taker* or carer*).tw,kw,kf. | 145452 |
| 8 | ((children* or son or sons or daughter* or offspring* or sibling* or brother* or sister* or wife* or wives or husband* or partner* or spous* or married* or famil* or parent* or father* or mother* or next of kin* or kinship* or significant other* or relative or relatives or informal or unpaid or old* or elder* or aged or aging or ageing or senior or seniors or geriatric) adj3 (care or caring or caregiving)).tw,kw,kf. | 136987 |
| 9 | Adult child/ or Sibling/ or First-degree relative/ or Nuclear family/ or Brother/ or Sister/ or Son/ or Daughter/ or Parent/ or Mother/ or Father/ or Family/ or Family relation/ or Child parent relation/ or Spouse/ or Domestic partner/ or Husband/ or Wife/ | 415233 |
| 10 | (caring or caregiving or (care adj1 giving)).ab. | 65432 |
| 11 | Care behavior/ | 7096 |
| 12 | 10 or 11 | 66647 |
| 13 | 9 and 12 | 11200 |
| 14 | (caring or caregiving or (care adj1 giving)).ti,kw,kf. | 21256 |
| 15 | 6 or 7 or 8 or 13 or 14 | 291986 |
| 16 | immigrant/ or migrant/ or emigrant/ | 28695 |
| 17 | refugee/ or asylum seeker/ | 15703 |
| 18 | undocumented immigrant/ | 602 |
| 19 | immigration/ or migration/ | 47684 |
| 20 | Minority group/ | 16829 |
| 21 | Ethnic group/ | 74762 |
| 22 | "ethnic or racial aspects"/ or ethnic difference/ or ethnicity/ or race difference/ or cultural diversity/ | 200411 |
| 23 | (Immigrant* or migrant* or multicultur* or intercultur* or minorit* or ethnic* or multiethnic* or racial* or refugee* or non western* or nonwestern* or asylum seeker*).tw,kw,kf. | 416585 |
| 24 | or/16-23 | 558185 |
| 25 | qualitative research/ | 101211 |
| 26 | hermeneutics/ | 673 |
| 27 | Interview/ | 230034 |
| 28 | narrative/ | 17916 |
| 29 | grounded theory/ | 8943 |
| 30 | Phenomenlology/ | 0 |
| 31 | Ethnography/ | 3299 |
| 32 | attitude/ | 67897 |
| 33 | behavior/ | 160327 |
| 34 | perception/ | 143090 |
| 35 | Experience/ | 38803 |
| 36 | (Qualitative or Narrative or Phenomenolog* or Hermeneutic* or interview* or Grounded theor* or Ethnograph* or themes or Attitude* or Behavior* or behaviour* or Perception* or View* or experience* or focus group*).tw,kw,kf. | 4628977 |
| 37 | or/25-36 | 4756416 |
| 38 | 5 and 15 and 24 and 37 | 2468 |
| 39 | limit 38 to yr="2021 -Current" | 329 |

**Database:** APA PsycInfo 1806 to June Week 3 2022

**Date:**  28.06.2022

**Results:** 132

|  | **Searches** | **Results** |
| --- | --- | --- |
| 1 | Older adulthood/ or dementia/ or Geriatrics/ | 60137 |
| 2 | ((aged or old*) adj2 (people or women or person* or men or immigrant* or minorit* or parent*)).tw. | 70820 |
| 3 | (senior or seniors or geriatric or elder or elders or elderly or dement* or aging or ageing or old age).tw. | 235693 |
| 4 | old*.ti,id. | 323007 |
| 5 | ("380" or "390").ag. | 365185 |
| 6 | or/1-5 | 727257 |
| 7 | Caregivers/ | 33449 |
| 8 | Caregiver burden/ | 6744 |
| 9 | (caregiver* or care giver* or caretaker* or care taker* or carer*).tw. | 72075 |
| 10 | ((children* or son or sons or daughter* or offspring* or sibling* or brother* or sister* or wife* or wives or husband* or partner* or spous* or married* or famil* or parent* or father* or mother* or next of kin* or kinship* or significant other* or relative or relatives or informal or unpaid or old* or elder* or aged or aging or ageing or senior or seniors or geriatric) adj3 (care or caring or caregiving)).tw. | 57924 |
| 11 | Adult offspring/ or Offspring/ or Sons/ or Daughters/ or Siblings/ or Brothers/ or Sisters/ or Family/ or Family relations/ or Nuclear family/ or Parents/ or mothers/ or fathers/ or Parent child relations/ or Spouses/ or Husbands/ or Wives/ or Significant others/ or Partners/ | 226050 |
| 12 | (caring or caregiving or (care adj1 giving)).ab. | 38838 |
| 13 | Caregiving/ | 7370 |
| 14 | 12 or 13 | 40451 |
| 15 | 11 and 14 | 8716 |
| 16 | (caring or caregiving or (care adj1 giving)).ti,id. | 14051 |
| 17 | 7 or 8 or 9 or 10 or 15 or 16 | 125166 |
| 18 | Immigration/ | 25253 |
| 19 | Refugees/ | 7570 |
| 20 | Human migration/ | 8537 |
| 21 | Minority Groups/ | 17208 |
| 22 | "racial and ethnic groups"/ | 14896 |
| 23 | Cultural Diversity/ | 2319 |
| 24 | Ethnic Identity/ | 18075 |
| 25 | Racial Identity/ | 2348 |
| 26 | Asylum seeking/ | 870 |
| 27 | (Immigrant* or migrant* or multicultur* or intercultur* or minorit* or ethnic* or multiethnic* or racial* or refugee* or non western* or nonwestern* or asylum seeker*).tw. | 228689 |
| 28 | or/18-27 | 239576 |
| 29 | qualitative methods/ | 10490 |
| 30 | focus group/ or focus group interview/ | 1016 |
| 31 | grounded theory/ | 4286 |
| 32 | narrative analysis/ | 1047 |
| 33 | semi-structured interview/ | 1254 |
| 34 | interviews/ | 11907 |
| 35 | qualitative measures/ | 95 |
| 36 | hermeneutics/ | 2373 |
| 37 | Phenomenology/ | 16711 |
| 38 | Ethnography/ | 10314 |
| 39 | attitudes/ | 29685 |
| 40 | Behavior/ | 27650 |
| 41 | Perception/ | 23599 |
| 42 | (Qualitative or Narrative or Phenomenolog* or Hermeneutic* or interview* or Grounded theor* or Ethnograph* or themes or Attitude* or Behavior* or bahaviour* or Perception* or View* or experience* or focus group*).tw. | 2370846 |
| 43 | or/29-42 | 2381728 |
| 44 | 6 and 17 and 28 and 43 | 1715 |
| 45 | limit 44 to yr="2021 -Current" | 132 |

**Database:** CINAHL with Full Text EBSCOhost

**Date:** 28.06.2026

**Results:** 165

| # | Query | Results |
| --- | --- | --- |
| S1 | (MH "Aged") OR (MH "Aged, 80 and Over") OR (MH "Frail Elderly") | 917,500 |
| S2 | (MH "Dementia") OR (MH "Dementia Patients") | 46,768 |
| S3 | (MH "Geriatrics") | 5,938 |
| S4 | TI ( ((aged or old*) N1 (people or women or person* or men or immigrant* or minorit* or parent*)) ) OR AB ( ((aged or old*) N1 (people or women or person* or men or immigrant* or minorit* or parent*)) ) | 101,950 |
| S5 | TI ( (senior or seniors or geriatric or elder or elders or elderly or dement* or aging or ageing or "old age") ) OR AB ( (senior or seniors or geriatric or elder or elders or elderly or dement* or aging or ageing or "old age") ) | 267,618 |
| S6 | TI old* | 111,005 |
| S7 | S1 OR S2 OR S3 OR S4 OR S5 OR S6 | 1,108,139 |
| S8 | (MH "Caregivers") OR (MH "Caregiver Burden") OR (MH "Caregiver Attitudes") OR (MH "Caregiver Support") | 51,225 |
| S9 | TI ( ( caregiver* or "care giver*" or caretaker* or "care taker*" or carer* ) ) OR AB ( ( caregiver* or "care giver*" or caretaker* or "care taker*" or carer* ) ) | 76,430 |
| S10 | (MH "Adult Children") OR (MH "Family") OR (MH "Family Relations") OR (MH "Nuclear Family") OR (MH "Daughters") OR (MH "Siblings") OR (MH "Sons") OR (MH "Spouses") OR (MH "Parents") | 125,555 |
| S11 | (MH "Patient-Family Relations") OR (MH "Fathers") OR (MH "Mothers") OR (MH "Significant Other") | 42,876 |
| S12 | S10 OR S11 | 161,879 |
| S13 | (MH "Caring") | 9,069 |
| S14 | AB ( caring or caregiving or (care N1 giving) ) | 43,273 |
| S15 | S13 OR S14 | 49,328 |
| S16 | S12 AND S15 | 7,915 |
| S17 | TI ( caring or caregiving or (care N1 giving) ) | 21,133 |
| S18 | TI ( ((children* or son or sons or daughter* or offspring* or sibling* or brother* or sister* or wife* or wives or husband* or partner* or spous* or married* or famil* or parent* or father* or mother* or "next of kin*" or kinship* or "significant other*" or relative or relatives or informal or unpaid or old* or elder* or aged or aging or ageing or senior or seniors or geriatric) N2 (care or caring or caregiving)) ) OR AB ( ((children* or son or sons or daughter* or offspring* or sibling* or brother* or sister* or wife* or wives or husband* or partner* or spous* or married* or famil* or parent* or father* or mother* or "next of kin*" or kinship* or "significant other*" or relative or relatives or informal or unpaid or old* or elder* or aged or aging or ageing or senior or seniors or geriatric) N2 (care or caring or caregiving)) ) | 80,458 |
| S19 | S8 OR S9 OR S16 OR S17 OR S18 | 171,448 |
| S20 | (MH "Immigrants") OR (MH "Immigrants, Illegal") OR (MH "Emigration and Immigration") | 22,402 |
| S21 | (MH "Minority Groups") OR (MH "Ethnic Groups") OR (MH "Cultural Diversity") OR (MH "Cultural Values") | 62,754 |
| S22 | (MH "Transients and Migrants") OR (MH "Undocumented Immigrants") OR (MH "Refugees") | 14,573 |
| S23 | TI ( ( Immigrant* or migrant* or multicultur* or intercultur* or minorit* or ethnic* or multiethnic* or racial* or refugee* or "non western*" or nonwestern* or "asylum seeker*" ) ) OR AB ( ( Immigrant* or migrant* or multicultur* or intercultur* or minorit* or ethnic* or multiethnic* or racial* or refugee* or "non western*" or nonwestern* or "asylum seeker*" ) ) | 138,126 |
| S24 | S20 OR S21 OR S22 OR S23 | 181,741 |
| S25 | (MH "Qualitative Studies") OR (MH "Action Research") OR (MH "Ethnographic Research") OR (MH "Ethnological Research") OR (MH "Ethnonursing Research") OR (MH "Grounded Theory") OR (MH "Naturalistic Inquiry") OR (MH "Phenomenological Research") OR (MH "Phenomenology") OR (MH "Focus Groups") OR (MH "Narratives") OR (MH "Interviews") OR (MH "Semi-Structured Interview") OR (MH "Structured Interview") OR (MH "Unstructured Interview") | 336,879 |
| S26 | (MH "Attitude") OR (MH "Family Attitudes") OR (MH "Caregiver Attitudes") OR (MH "Behavior") OR (MH "Perception") | 78,084 |
| S27 | TI ( ( Qualitative or Narrative or Phenomenolog* or Hermeneutic* or interview* or "Grounded theor*" or Ethnograph* or themes or Attitude* or Behavior* or Behaviour* or Perception* or View* or experience* or "focus group*" ) ) OR AB ( ( Qualitative or Narrative or Phenomenolog* or Hermeneutic* or interview* or "Grounded theor*" or Ethnograph* or themes or Attitude* or Behavior* or Behaviour* or Perception* or View* or experience* or "focus group*" ) ) | 1,206,788 |
| S28 | S25 OR S26 OR S27 | 1,316,070 |
| S29 | S7 AND S19 AND S24 AND S28 | 1,840 |
| S30 | S7 AND S19 AND S24 AND S28  Limiters - Published Date: 20210101-20221231 | 165 |

**Database:** SocIndex EBSCOhost

**Date:** 28.06.2026

**Results:** 92

| # | Query | Results |
| --- | --- | --- |
| S1 | DE "OLD age -- Social aspects" OR DE "OLDER women" OR DE "OLDER men" OR DE "SENILE dementia" OR DE "OLDER parents" OR DE "AGING parents" OR DE "MINORITY older people" OR DE "OLDER immigrants" OR DE "OLDER people" OR DE "CROSS cultural studies on older people" OR DE "OLD age" OR DE "AGING" OR DE "GERIATRICS" OR DE "ELDER care" OR DE "HOME care of older people" | 32,471 |
| S2 | TI ( ((aged or old*) N1 (people or women or person* or men or immigrant* or minorit* or parent*)) ) OR AB ( ((aged or old*) N1 (people or women or person* or men or immigrant* or minorit* or parent*)) ) OR KW ( ((aged or old*) N1 (people or women or person* or men or immigrant* or minorit* or parent*)) ) OR SU ( ((aged or old*) N1 (people or women or person* or men or immigrant* or minorit* or parent*)) ) | 42,007 |
| S3 | TI ( (senior or seniors or geriatric or elder or elders or elderly or dement* or aging or ageing or "old age") ) OR AB ( (senior or seniors or geriatric or elder or elders or elderly or dement* or aging or ageing or "old age") ) OR KW ( (senior or seniors or geriatric or elder or elders or elderly or dement* or aging or ageing or "old age") ) OR SU ( (senior or seniors or geriatric or elder or elders or elderly or dement* or aging or ageing or "old age") ) | 80,153 |
| S4 | TI old* OR KW old* | 31,441 |
| S5 | S1 OR S2 OR S3 OR S4 | 107,212 |
| S6 | DE "MALE caregivers" OR DE "WOMEN caregivers" | 150 |
| S7 | DE "CAREGIVERS -- Social aspects" OR DE "PSYCHOLOGY of caregivers" OR DE "KINSHIP care" OR DE "HEALTH of caregivers" OR DE FAMILY relationships of caregivers | 1,576 |
| S8 | TI ( caregiver* or "care giver*" or caretaker* or "care taker*" or carer* ) OR AB ( caregiver* or "care giver*" or caretaker* or "care taker*" or carer* ) OR KW ( caregiver* or "care giver*" or caretaker* or "care taker*" or carer* ) OR SU ( caregiver* or "care giver*" or caretaker* or "care taker*" or carer* ) | 21,102 |
| S9 | DE "FAMILIES" OR DE "FAMILY relations" OR DE "FAMILY relationships of older people" OR DE "ADULT children" OR DE "ADULT children of aging parents" OR DE "PARENT & adult child" OR DE "ADULT children living with parents" OR DE "FAMILY relationships of adult children of aging parents" OR DE "ADULT children family relationships" | 40,369 |
| S10 | DE "DAUGHTERS" OR DE "NUCLEAR families" OR DE "PARENTS" OR DE "SIBLINGS" OR DE "SONS" OR DE "BROTHERS" OR DE "SISTERS" OR DE "HUSBANDS" OR DE "WIVES" OR DE "SIGNIFICANT others" OR DE "HUSBAND & wife" OR DE "MARRIED people" OR DE "SPOUSES" OR DE "MARITAL relations" OR DE "mothers" OR DE "fathers" OR DE "OLDER parents" OR DE "AGING parents" | 31,406 |
| S11 | DE "MINORITY families" OR DE "MINORITY parents" OR DE "CHILDREN of minorities" OR DE "ADULT children of immigrants" OR DE "CHILDREN of immigrants" | 1,264 |
| S12 | S9 OR S10 OR S11 | 66,936 |
| S13 | DE "CARE of people" OR DE "ELDER care" | 5,819 |
| S14 | DE "CARING" OR DE "HOME care of older people" | 2,452 |
| S15 | AB ( caring or caregiving or (care N1 giving) ) | 13,285 |
| S16 | S13 OR S14 OR S15 | 19,862 |
| S17 | S12 AND S16 | 2,641 |
| S18 | TI ( caring or caregiving or (care N1 giving) ) OR KW ( caring or caregiving or (care N1 giving) ) | 5,801 |
| S19 | TI ( ((children* or son or sons or daughter* or offspring* or sibling* or brother* or sister* or wife* or wives or husband* or partner* or spous* or married* or famil* or parent* or father* or mother* or "next of kin*" or kinship* or "significant other*" or relative or relatives or informal or unpaid or old* or elder* or aged or aging or ageing or senior or seniors or geriatric) N2 (care or caring or caregiving)) ) OR AB ( ((children* or son or sons or daughter* or offspring* or sibling* or brother* or sister* or wife* or wives or husband* or partner* or spous* or married* or famil* or parent* or father* or mother* or "next of kin*" or kinship* or "significant other*" or relative or relatives or informal or unpaid or old* or elder* or aged or aging or ageing or senior or seniors or geriatric) N2 (care or caring or caregiving)) ) OR KW ( ((children* or son or sons or daughter* or offspring* or sibling* or brother* or sister* or wife* or wives or husband* or partner* or spous* or married* or famil* or parent* or father* or mother* or "next of kin*" or kinship* or "significant other*" or relative or relatives or informal or unpaid or old* or elder* or aged or aging or ageing or senior or seniors or geriatric) N2 (care or caring or caregiving)) ) OR SU ( ((children* or son or sons or daughter* or offspring* or sibling* or brother* or sister* or wife* or wives or husband* or partner* or spous* or married* or famil* or parent* or father* or mother* or "next of kin*" or kinship* or "significant other*" or relative or relatives or informal or unpaid or old* or elder* or aged or aging or ageing or senior or seniors or geriatric) N2 (care or caring or caregiving)) ) | 32,242 |
| S20 | S6 OR S7 OR S8 OR S17 OR S18 OR S19 | 49,122 |
| S21 | DE "MINORITY older people" OR DE "MINORITIES" OR DE "IMMIGRANT men" OR DE "REFUGEES" OR DE "UNDOCUMENTED immigrants" OR DE "UNDOCUMENTED immigrants -- Medical care" OR DE "IMMIGRANTS -- Medical care" OR DE "OLDER immigrants" OR DE "CROSS cultural studies on older people" OR DE "MINORITY families" OR DE "MINORITY parents" OR DE "CHILDREN of minorities" OR DE "ADULT children of immigrants" OR DE "CHILDREN of immigrants" | 19,954 |
| S22 | DE "PERMANENT residents (Immigrants)" OR DE "WOMEN immigrants" OR DE "IMMIGRANTS" OR DE "EMIGRATION & immigration" | 40,089 |
| S23 | DE "ETHNIC groups" OR DE "ETHNICITY" OR DE "ATTITUDES of ethnic groups" OR DE "CROSS-cultural differences" OR DE "ETHNIC differences" OR DE "CULTURAL pluralism" OR DE "CULTURAL values" OR DE "RACIAL differences" OR DE "RACIAL minorities" OR DE "HEALTH & race" OR DE "WOMEN refugees" OR DE "MEDICAL care of refugees" OR DE "ETHNIC differences" | 40,076 |
| S24 | TI ( Immigrant* or migrant* or multicultur* or intercultur* or minorit* or ethnic* or multiethnic* or racial* or refugee* or "non western*" or nonwestern* or "asylum seeker*" ) OR AB ( Immigrant* or migrant* or multicultur* or intercultur* or minorit* or ethnic* or multiethnic* or racial* or refugee* or "non western*" or nonwestern* or "asylum seeker*" ) OR KW ( Immigrant* or migrant* or multicultur* or intercultur* or minorit* or ethnic* or multiethnic* or racial* or refugee* or "non western*" or nonwestern* or "asylum seeker*" ) OR SU ( Immigrant* or migrant* or multicultur* or intercultur* or minorit* or ethnic* or multiethnic* or racial* or refugee* or "non western*" or nonwestern* or "asylum seeker*" ) | 223,674 |
| S25 | S21 OR S22 OR S23 OR S24 | 238,300 |
| S26 | DE "QUALITATIVE research" OR DE "CONVERSATION analysis" OR DE "FOCUS groups" OR DE "INTERVIEWING" | 37,059 |
| S27 | DE "HERMENEUTICS" OR DE "EXPERIENCE" OR DE "PHENOMENOLOGY" OR DE "ATTITUDE (Psychology)" OR DE "BEHAVIOR" OR DE "HUMAN behavior" OR DE "PERCEPTION" | 58,026 |
| S28 | TI ( Qualitative or Narrative or Phenomenolog* or Hermeneutic* or interview* or "Grounded theor*" or Ethnograph* or themes or Attitude* or Behavior* or Behaviour* or Perception* or View* or experience* or "focus group*" ) OR AB ( Qualitative or Narrative or Phenomenolog* or Hermeneutic* or interview* or "Grounded theor*" or Ethnograph* or themes or Attitude* or Behavior* or Behaviour* or Perception* or View* or experience* or "focus group*" ) OR KW ( Qualitative or Narrative or Phenomenolog* or Hermeneutic* or interview* or "Grounded theor*" or Ethnograph* or themes or Attitude* or Behavior* or Behaviour* or Perception* or View* or experience* or "focus group*" ) OR SU ( Qualitative or Narrative or Phenomenolog* or Hermeneutic* or interview* or "Grounded theor*" or Ethnograph* or themes or Attitude* or Behavior* or Behaviour* or Perception* or View* or experience* or "focus group*" ) | 821,638 |
| S29 | S26 OR S27 OR S28 | 822,178 |
| S30 | S5 AND S20 AND S25 AND S29 | 1,077 |
| S31 | S5 AND S20 AND S25 AND S29 Limiters - Date of Publication: 20210101-20221231 | 92 |

**Database:** Social Care Online

**Date:** 28.06.2022

**Results:** 25

- SubjectTerms:'"carers"' including this term only

- OR AllFields:'carer*'

- OR AllFields:'caregiv*'

- OR AllFields:'"care giver"'

- OR AllFields:'"care givers"'

- OR AllFields:'caretaker*'

- OR AllFields:'family'

- OR AllFields:'families'

AND

- SubjectTerms:'"migrants"' including this term only

- OR SubjectTerms:'"migration"' including this term only

- OR SubjectTerms:'"immigration"' including this term only

- OR SubjectTerms:'"black and minority ethnic people"' including this term only

- OR SubjectTerms:'"immigrants"' including this term only

- OR SubjectTerms:'"ethnicity"' including this term only

- OR SubjectTerms:'"cultural identity"' including this term only

- OR SubjectTerms:'"asylum seekers"' including this term only

- OR SubjectTerms:'"refugees"' including this term only

- OR AllFields:'immigra*'

- OR AllFields:'migra*'

- OR AllFields:'ethnic*'

- OR AllFields:'minorit*'

- OR AllFields:'multiethnic*'

- OR AllFields:'refugee*'

- OR AllFields:'asylum seeker*'

AND

- SubjectTerms:'"qualitative research"' including this term only

- OR SubjectTerms:'"interviewing"' including this term only

- OR SubjectTerms:'"focus groups"' including this term only

- OR SubjectTerms:'"attitudes"' including this term only

- OR SubjectTerms:'"behaviour"' including this term only

- OR AllFields:'qualitative'

- OR AllFields:'interview*'

- OR AllFields:'"focus group"'

- OR AllFields:'"focus groups"'

- OR AllFields:'experience*'

AND

- SubjectTerms:'"older people"' including this term only

- OR SubjectTerms:'"dementia"' including this term only

- OR SubjectTerms:'"ageing"' including this term only

- OR AllFields:'aged'

- OR AllFields:'aging'

- OR AllFields:'ageing'

- OR AllFields:'old*'

- OR AllFields:'elder*'

AND

- PublicationYear:'2021 2023'

**Database:** Applied Social Sciences Index & Abstracts (ASSIA) via ProQuest

**Date:** 28.06.2022

**Results:** 75

[STRICT] (noft((aged OR old*) NEAR/1 (people OR women OR person* OR men OR immigrant* OR minorit* OR parent*)) OR noft((senior OR seniors OR geriatric OR elder OR elders OR elderly OR dement* OR aging OR ageing OR "old age")) OR ti(old*)) AND (noft(caregiver* OR "care giver*" OR caretaker* OR "care taker*" OR carer*) OR noft((children* OR son OR sons OR daughter* OR offspring* OR sibling* OR brother* OR sister* OR wife* OR wives OR husband* OR partner* OR spous* OR married* OR famil* OR parent* OR father* OR mother* OR "next of kin*" OR kinship* OR "significant other*" OR relative OR relatives OR informal OR unpaid OR old* OR elder* OR aged OR aging OR ageing OR senior OR seniors OR geriatric) NEAR/2 (care OR caring OR caregiving)) OR ti(caring OR caregiving OR (care NEAR/1 giving)) OR su(caring OR caregiving OR (care NEAR/1 giving))) AND noft(Immigrant* OR migrant* OR multicultur* OR intercultur* OR minorit* OR ethnic* OR multiethnic* OR racial* OR refugee* OR "non western*" OR nonwestern* OR "asylum seeker*") AND noft(Qualitative OR Narrative OR Phenomenolog* OR Hermeneutic* OR interview* OR "Grounded theor*" OR Ethnograph* OR themes OR Attitude* OR Behavior* OR Behaviour* OR Perception* OR View* OR experience* OR "focus group*") Limited to 2021-2023

**Database:** Scopus via Elsevier

**Date:** 28.06.2022

**Results:** 250

( ( TITLE-ABS-KEY ( ( aged OR old* ) W/1 ( people OR women OR person* OR men OR immigrant* OR minorit* OR parent* ) ) OR TITLE-ABS-KEY ( ( senior OR seniors OR geriatric OR elder OR elders OR elderly OR dement* OR aging OR ageing OR "old age" ) ) OR TITLE ( old* ) OR KEY ( old* ) ) ) AND ( ( TITLE-ABS-KEY ( ( caregiver* OR "care giver*" OR caretaker* OR "care taker*" OR carer* ) ) OR TITLE-ABS-KEY ( ( children* OR son OR sons OR daughter* OR offspring* OR sibling* OR brother* OR sister* OR wife* OR wives OR husband* OR partner* OR spous* OR married* OR famil* OR parent* OR father* OR mother* OR "next of kin*" OR kinship* OR "significant other*" OR relative OR relatives OR informal OR unpaid OR old* OR elder* OR aged OR aging OR ageing OR senior OR seniors OR geriatric ) W/2 ( care OR caring OR caregiving ) ) OR TITLE ( caring OR caregiving OR ( care W/0 giving ) ) OR KEY ( caring OR caregiving OR ( care W/0 giving ) ) ) ) AND ( TITLE-ABS-KEY ( immigrant* OR migrant* OR multicultur* OR intercultur* OR minorit* OR ethnic* OR multiethnic* OR racial* OR refugee* OR "non western*" OR nonwestern OR "asylum seeker*" ) ) AND ( ( TITLE-ABS-KEY ( qualitative OR narrative OR phenomenolog* OR hermeneutic* OR interview* OR "grounded theor*" OR ethnograph* OR themes OR "focus group*" ) OR TITLE-ABS-KEY ( ( attitude* OR behavior* OR perception* OR view* OR experience* ) W/2 ( immigrant* OR migrant* OR multicultur* OR intercultur* OR minorit* OR ethnic* OR multiethnic* OR racial* OR refugee* OR "non western*" OR nonwestern* OR "asylum seeker*" OR caregiver* OR "care giver*" OR caretaker* OR "care taker*" OR carer* ) ) ) ) AND ( LIMIT-TO ( PUBYEAR , 2022 ) OR LIMIT-TO ( PUBYEAR , 2021 ) )

**Database:** Google Scholar (Publish & Perish version 7.15.2643.7260)

**Date:** 29.06.2022

**Results:** 150

older|elder|elderly|aging|ageing|"old people|men|women|immigrants|parents"|dementia "family caregiver|caregivers" immigrant|immigrants|migrant|migrants|ethnic qualitative|interview|interviews|"focus groups"
